# Matching implementation strategies to barriers and facilitators for a lifestyle front office in the hospital: a qualitative study

**DOI:** 10.1186/s12913-025-13452-8

**Published:** 2025-09-30

**Authors:** Marlinde Lianne van Dijk, Joyce Vrijsen, Leonie Mariëlle te Loo, Inge van den Akker-Scheek, Martine de Bruijne, Rienk Dekker, Willem van Mechelen, Femke van Nassau, Judith G.M. Jelsma

**Affiliations:** 1 Department of Public and Occupational Health, Amsterdam UMC, Location Vrije Universiteit Amsterdam, De Boelelaan 1117, Amsterdam, The Netherlands; 2 Health Behaviors & Chronic Diseases, Amsterdam Public Health Research Institute, Amsterdam, The Netherlands; 3 Quality of Care, Amsterdam Public Health Research Institute, Amsterdam, The Netherlands; 4 Department of Orthopaedics, University of Groningen, University Medical Center Groningen, Groningen, The Netherlands; 5 Centre of Expertise Prevention in Health and Social Care, Faculty of Health, Sports and Social Work, Inholland University of Applied Sciences, Haarlem, The Netherlands; 6 Department of Rehabilitation Medicine, University of Groningen, University Medical Center Groningen, Groningen, The Netherlands

**Keywords:** Lifestyle front office, Behavioural change, Prevention, Hospital, Qualitative, Implementation

## Abstract

**Background:**

In Dutch hospitals, advice on healthy lifestyle during consultation with healthcare professionals is hindered by limited time, insufficient skills and limited knowledge on referral options. In order to organize a new care pathway in which care related to healthy lifestyle is provided through a dedicated lifestyle front office (LFO) in the hospital, implementation barriers and facilitators were identified and matched to tailored implementation strategies.

**Methods:**

Semi-structured interviews were held between March and August 2021 with healthcare professionals (i.e. specialists, physician assistants, dieticians, physiotherapist, (specialized) nurses) from different clinical departments (*n* = 33), and with patients (*n* = 27) diagnosed with a non-communicable disease (NCD) that were treated in out-patient clinics of the hospital and had a body mass index of 25 ≥ kg/m^2^ and/or were current smokers. An inductive thematic analysis was conducted to identify barriers and facilitators for implementation. Barriers were matched to implementation strategies with the CFIR-ERIC Implementation Strategy Matching Tool and further operationalized for use in practice.

**Results:**

Barriers and facilitators were clustered according to different organizational stages of the identified care pathway. Referral to LFO includes six topics: healthcare professionals’ beliefs about lifestyle; patient motivation for lifestyle change; referral skills and knowledge of healthcare professionals; digital resource support for referral; feedback after referral; and responsibility for referral. Appointment at LFO was affected by six topics: financial burden of the additional visit; time, skills and knowledge of lifestyle broker; physical location of LFO; efficiency in care planning; fragmentation; and prevention as task of the general practitioner. Regarding referral to community-based lifestyle initiatives four barriers were identified: financial burden of community-based lifestyle initiative; geographical availability; quality assurance of community-based lifestyle initiatives; and collaboration. Implementation strategies included building an infrastructure, creating a learning collaborative, preparing a referral tool, identifying local champions, informing stakeholders, conducting training, building a coalition, collecting testimonials and accessing new funding.

**Conclusions:**

Insights from the current qualitative study were based on a large and diverse stakeholder group and provided important insights for the implementation of an LFO in the hospital. Future research should provide information on effectiveness of actual implementation of the implementation strategies in an LFO in the hospital.

**Supplementary Information:**

The online version contains supplementary material available at 10.1186/s12913-025-13452-8.

## Background

The increase in the prevalence of lifestyle-related non-communicable diseases (NCDs), such as cardiovascular disease, diabetes or cancer, remains a major challenge for many countries worldwide. On a global level, 71% of all deaths can be accounted for by NCDs [1, 2]. The Netherlands is no exception: in 2019 it was estimated that 57% of the Dutch population had at least one NCD [3]. The manifestation and exacerbation of NCDs has been largely attributed to modifiable behaviours that relate to an unhealthy lifestyle, such as tobacco use, unhealthy diet, physical inactivity and alcohol intake [2, 4]. These modifiable risk factors are a target for policy makers and governing bodies in their pursuit to improve public health [5]. Attention for prevention becomes even more important due to ageing populations, that will result in an increase in the volume of patients in need of healthcare and a decline in the available workforce to provide this care.

Lifestyle interventions aimed at lifestyle improvement are compelling, as they have effects that are beneficial for overall health and may be equally effective as drug interventions for some chronic conditions [6–9]. Additionally, a healthy lifestyle contributes to the prevention of developing additional NCDs and multimorbidity [10]. Integrating lifestyle in the treatment of patients provides a strong ‘window of opportunity’ for lifestyle change, in particular for hospital patients living with an NCD. This specific group is seen by a multitude of healthcare professionals who provide treatment and care. Therefore, introducing a tailored lifestyle intervention in addition to medical care as usual (e.g. surgery, medication) can be beneficial for both the patient and the professional [11]. Despite clinical guidelines that include lifestyle improvement as part of treatment of patients, and a wide availability of lifestyle interventions in the community, promoting a healthy lifestyle is not yet widely implemented in clinical care in the Netherlands [12]. This lack of integration of lifestyle interventions as a standard option of choice in individual treatment plans is related to time constraints, insufficient skills and personal preferences of healthcare professionals [13–17].

To incorporate lifestyle promotion in clinical care, and to deal with the aforementioned barriers experienced by healthcare professionals, we wanted to develop a lifestyle front office (LFO). This LFO should help to guide and motivate patients who are already under treatment at the hospital, to make lifestyle changes and to refer patients to community-based lifestyle initiatives for follow-up care. However, implementing innovative concepts in clinical healthcare is challenging, due to specific clinical organization culture and restricted resources [14, 18]. Effective healthcare delivery relies not merely on evidence-based interventions but also on the implementation of tailored strategies that address the unique needs of specific populations and care settings. Customizing the approach ensures that innovations are adopted and sustained, leading to improved patient outcomes and more equitable access to care [19]. Implementation science offers structured approaches to identify barriers and adapt interventions to local contexts [20]. Therefore, the aim of this study was to identify implementation barriers and facilitators from both the perspectives of patients and healthcare professionals in the outpatient clinic of the hospital and to use these study results to develop matching implementation strategies. These implementation strategies should facilitate optimal integration and implementation of the LFO in the future.

## Methods

### Context – healthcare system and concept of a lifestyle front office

This study was done preceding the development of the LFO in two university hospitals in the Netherlands. The initial idea of the LFO was that patients are referred by their healthcare professionals to the LFO. In the LFO, patients would have a first consultation with a lifestyle broker (LSB), who is skilled in lifestyle counselling and who uses motivational interviewing (MI) [21, 22] to support patients in building motivation towards lifestyle change, before exploring and referring to established community-based lifestyle interventions (e.g. smoking cessation program, lifestyle coach, dietician). In the Dutch healthcare system, high-quality healthcare is accessible to all and is financed through a system of solidarity, combining income-based taxation with mandatory health insurance [23]. Which services are covered under the mandatory basic insurance package is decided on by the government, as well as corresponding responsibilities that are assigned to various stakeholders in the system (e.g. health insurers, municipalities, organisations, professionals, and citizens) [24]. Within this framework, the government aims to create optimal conditions for public health, while individuals are expected to take responsibility for their own health where possible. When necessary, people can seek professional care, and access to specialized services is available upon referral. Community-based lifestyle initiatives can be (partly) reimbursed within the basic or additional insurance packages but can differ per type, amount of sessions and/or previously used healthcare. Availability of initiatives may differ per region (i.e. rural vs. city) and type (e.g. lifestyle programs with maximum amount of participants vs. online smoking cessation course). Implementation of an LFO from the perspective of healthcare professionals working in community-based lifestyle initiatives is described elsewhere [25].

### Study design

This qualitative study consisted of two phases. Phase 1 focused on exploring potential barriers and facilitators for implementation of an LFO in a clinical setting. Phase 2 focused on matching implementation strategies to the established barriers and facilitators, in order to design an implementation plan. Interviews were performed with patients and healthcare professionals to gain insight in their views, needs and preferences on the design and implementation of an LFO. The COREQ guideline (Appendix I) was used to ensure complete reporting [26]. The study was exempted from review by the medical ethical committee of Amsterdam UMC (2020.0756).

### Study population and sampling

The study population consisted of healthcare professionals working in clinical and outpatient care, and patients with an NCD. Patients were recruited through healthcare professionals and through social media by researchers and research interns. They were contacted through email to make an appointment for an interview. Patients were included if they were at risk of/diagnosed with an NCD (i.e. osteoarthritis, diabetes, cardiovascular disease) and were overweight (Body Mass Index (BMI) of 25 kg/m^2^or higher) and/or were a smoker. Healthcare professionals were recruited by email sent to the following departments from two university medical centres in the Netherlands: cardiology, internal medicine, neurology, and orthopaedics. Due to the nature of the recruitment method, there is no data on the number of patients and healthcare professionals who have been approached nor on refusal of participation.

### Data collection

Due to nationwide COVID-19 restrictions, interviews were conducted by video call (Microsoft Teams©) or by telephone. A semi-structured topic guide was used during the interviews (appendix II and III), which was inspired by previous experience and implementation research on determinants by Flottorp et al. [27]. At the start of the interview the study’s purpose and concept of an LFO were explained. Patients were asked about needs, views and preferences regarding lifestyle guidance and development of an LFO. All patients participated from their home; in one interview the partner participated as well. Healthcare professionals were interviewed to assess their current experiences with lifestyle guidance and referral in clinical and outpatient care, as well as views and preferences regarding implementation of an LFO. Most healthcare professionals were interviewed while they were physically at work, with the exception of a few who were interviewed in the evening and participated from home.

Data collection took place between March and August 2021. Each participant received written information on the study and the procedure. Written informed consent was obtained from every participant. Interviews were done by JJ, JV, MvD, LvS, CJ, SZ and EK, who were working respectively as senior researcher (JJ, JV), PhD-student (MvD) and research intern (LvS, CJ, SZ, EK). All were female and trained in and experienced with doing interviews. Educational background included human movement sciences, health sciences and nursing. One interviewer (JJ) and an involved senior researcher (FN) had extensive experience with implementation research and were involved in the design of the study. Interviews were expected to last between 45 and 60 min. All interviews were audio-recorded, and audio files were transcribed by an external company (i.e. Amberscript) [28]. Field notes were made during interviews.

### Data analysis

#### Phase 1 – Barriers and facilitators for implementation

Thematic analysis was conducted in Atlas.ti version 8. Thematic analysis is a method of identifying, analysing and reporting patterns (themes) within data following five steps: (1) compiling (i.e. collecting, familiarization and transcription of data); (2) disassembling (i.e. initial open coding of the data with Atlas.ti); (3) reassembling (i.e. creating a codebook with (sub)themes); (4) interpreting (i.e. re-reading of all codes within themes and creating analytical conclusions clustered accordingly; 5) concluding (i.e. creating answers to the research question(s)) [29]. Analyses were carried out after all data were collected. The entire dataset was used for analysis and no separate analyses were done for patients or healthcare professionals. Investigator triangulation was ensured by having a clear interview guide and protocol, training on the protocol, various peer debriefing sessions, and having multiple coders (JJ, JV, MvD) conducting the inductive analysis to reach intercoder agreement.

#### Phase 2 – Implementation strategies

In order to find tailored implementation strategies, we used the Consolidated Framework for Implementation Research (CFIR) [30]. This framework offers a comprehensive structure for understanding and guiding the implementation of complex interventions in healthcare settings. By organizing key constructs into five major domains, CFIR enables researchers and practitioners to systematically assess contextual factors that influence implementation success [30]. Using CFIR provided a pragmatic way to address real-world concepts while still using evidence-based implementation theory. Barriers from phase 1 were linked to the CFIR constructs and then added to the ERIC Implementation Strategy Matching Tool [30, 31]. This tool combines concepts of CFIR with implementation strategies derived from the Expert Recommendations for Implementing Change (ERIC) project [31]. For this phase, we matched barriers to the CFIR constructs from the following domains: intervention characteristics, outer setting, inner setting, characteristics of individuals, and process. We then put our barriers into the CFIR-ERIC matching tool to score all constructs and different combinations of constructs. The output of the matching tool is a set of implementation strategies, ranked on a cumulative percentage value. This percentage represents the value of the match between topic and strategy according to the ERIC experts [30, 31]. We selected the top four matching implementation strategies. The selected strategies were then further operationalized with stakeholder suggestions (e.g. *the barrier ‘attitude towards effectiveness of lifestyle interventions’ was matched to CFIR topic ‘Evidence strength & Quality’*,* of which the tool suggested the ERIC strategy ‘Conduct educational meetings’. This was operationalized into ‘joining already planned educational sessions of healthcare professionals and present the study’*). After formulating the operationalisation of the implementation strategies, we identified required resources and matched all strategies to a timeline. In this phase investigator triangulation was ensured by having multiple researchers (JJ, MvD, JV) carry out all activities. Implementation strategies were presented and discussed within the LOFIT consortium with representation of experts with different expertise.

## Results

### Characteristics

In total 60 participants were interviewed, of which 27 were patients and 33 were healthcare professionals employed by one of the two university medical centres. A description of the population can be found in Table 1a and 1b. Nearly two thirds of the patients were women (63.0%) and age ranged between 34 and 88 years, with a mean of 59.0 years. About half of the patients had received higher education (51.9%). The larger part of patients had CVD (70.4%) and the remaining patients were diagnosed with osteoarthritis (29.6%). Of the healthcare professionals, slightly more than half (55.5%) was women; age ranging from 27 to 66 years, with a mean of 43.0 years. They were mostly working at the department of internal medicine (60.6%), followed by orthopaedics (21.2%). The healthcare professionals represented a diverse job profile, with the majority working as a medical specialist (42.2%). Working experience in current function ranged from one to 30 years, with a mean of 9.6 years.

**Table 1a:** Characteristics of patients

	Total *N* = 27
Gender (*N*,%)	
Man	10 (37.0)
Woman	17 (63.0)
Age, mean (SD; range), years	59.0 (14.5; 34.0–88.0)
Education level^1^ (*N*, %)	
Lower	4 (14.8)
Medium	9 (33.3)
High	14 (51.9)
Medical condition (*N*, %)	
CVD^2^	19 (70.4)
Osteoarthritis	8 (29.6)

^1^Education level: lower = no formal qualification, elementary education and/or pre-vocational education; medium = vocational education ; high = university (of applied sciences) education

^2^CVD = cardiovascular disease

**Table 1b:** Characteristics of healthcare professionals

	Total *N* = 33
Gender (*N*,%)	
Man	15 (45.5)
Woman	18 (55.5)
Age, mean (SD; range), years	43.7 (10.4; 27.0–66.0)
Department	(*N*, %)
Internal medicine	20 (60.6)
Orthopaedics	7 (21.2)
Cardiology	3 (9.1)
Neurology	3 (9.1)
Job title healthcare professional	(*N*, %)
Medical Specialist	14 (42.2)
Nurse (consultant)	5 (15.2)
Physiotherapist	4 (12.1)
Nursing practitioner	3 (9.1)
Resident	3 (9.1)
Dietician	2 (6.1)
Physician assistant	1 (3.0)
Psychologist	1 (3.0)
Work experience, (mean, range) years	9.6 (8.0 ; 1.0–30.0)
Work experience (categories)	(N,%)
<5 year	11 (34.4)
5–9 year	10 (31.3)
>10 years	11 (34.14)

### Barriers and facilitators

Barriers and facilitators are presented in the context of the three stages of the LFO care pathway: (1) referral to the LFO by the healthcare professional; (2) care delivery at LFO; (3) referral to community-based lifestyle initiative.

### Stage 1: Referral to LFO by the healthcare professional in the outpatient hospital clinic

#### Healthcare professionals’ beliefs about lifestyle

The beliefs about lifestyle as part of clinical treatment pertained different expectations regarding effectiveness and long-term effects among healthcare professionals. Positive beliefs may work as an implementation facilitator, and negative beliefs as barrier to referral. Some healthcare professionals expressed that their perceived need of lifestyle-as-medicine as part of healthcare motivated them to refer to the LFO. Past experiences with discussing lifestyle change with patients, positive or negative, also functioned as barrier or facilitator for referral to the LFO. Negative experiences with similar implementation projects in the past were mentioned as barrier. Some healthcare professionals thought of lifestyle change as a burden and preferred to prescribe drugs in order to achieve the same goals.

I think this is a great initiative. If, as a hospital organization, you do not take action or fail to organize anything you miss a great opportunity, I think. It’s an important opportunity to improve the situation of many patients – Specialist (#4, orthopaedics, 55 years old)

I do not have a high opinion on it [lifestyle-as-medicine], because I think it is very ineffective, actually not effective at all. I am pretty negative about it. – Resident (#24, internal medicine, 32 years old)

#### Patient motivation for lifestyle change

Many healthcare professionals mentioned to be discouraged to refer patients to the LFO if the patient was not motivated. Beliefs about patients who were unmotivated or not suited included patients with a limited life expectancy or those of older age; with insufficient skills and knowledge; with past negative experiences regarding lifestyle change; who were already included in a different research population; who did not want an additional professional; who were ashamed of their current lifestyle; and, who preferred drugs over a lifestyle intervention.

I think the very first question is, and I do this myself at the outpatient clinics: are you open to listening to a brief talk about smoking cessation? If the answer is no, than I won’t continue– Resident (#24, internal medicine, 32 years old)

For some healthcare professionals, unmotivated patients were seen as a sign to provide more information so they could consider lifestyle and be referred at a later point in time. Likewise, motivated patients were a facilitator for referral to the LFO by healthcare professionals. For patients, facilitators for going to the LFO included if their healthcare professional advised to do so; if their healthcare professional had no answers to their lifestyle-related questions; and if referral to the LFO was favouring their condition and treatment.

I assume you will be referred. And if the doctor says so, than that’s what you will do, because I assume that is a well-considered choice and that you can benefit from it. Especially when you have problems and you don’t know what to do. – Patient (#37, CVD, 52 years old)

#### Referral skills and knowledge of healthcare professionals

If healthcare professionals felt they were skilled and knowledgeable enough on lifestyle change and the referral protocol, this would facilitate them in referring patients. Additionally, it would facilitate them if there was a reason to discuss lifestyle change and the referral to the LFO (e.g. lab results, introduction of LFO in hospital care).

Generally you see that in a short consultation it is quite difficult. I find it a tricky topic. I think that has to do with my age and the fact that I don’t always feel confident in that at the moment I have to tell someone [to live more healthy]. It’s always tricky anyway when you have to get someone to be physically active independently, instead of giving them a solution. People want an easy, quick solution, but of course there often isn’t one. People are a little disappointed with that at first anyway. – Physician assistant (#9, orthopaedics, 30 years old)

#### Digital resource support for referral

All healthcare professionals deemed digital resource support crucial for implementation. If the Electronic Patient File (EPF) of the hospital can be used to integrate the new referral protocols, this would be beneficial for both patients and healthcare professionals. Two healthcare professionals mentioned specific features that should be added, such as a list of lifestyle behaviours that can be checked and a checkbox or pop-up if a patient would be eligible for referral.

[.] and the support, like we receive from the lab and radiology. [If we could use that] that would be fantastic of course. – Specialist (#20, internal medicine, 42 years old)

#### Feedback after referral

After referral to the LFO, healthcare professionals would appreciate feedback by a professional in the LFO in the EPF on the status and progress of the patient (e.g. what happened after referral to the LFO, lifestyle intervention of choice). Insight on patient status after referral was mentioned by some healthcare professionals as a facilitator to refer more patients in the future.

Feedback towards us is a good idea. It will give us an impression how it’s going and we can provide support when it’s not going well or something like that. – Nurse practitioner (#23, internal medicine, 29 years old)

In fact, I think that feedback also works well in order to refer people back to the lifestyle front office. If you know: “oh, yes, this is how this works” or “that is the feeling”, then I think the healthcare professionals are also more likely to refer.” – Psychologist (#26, internal medicine, 54 years old)

#### Responsibility for referral

Many healthcare professionals expressed that referral should be open to all types of healthcare professionals, as the patient population of an academic hospital is known for complex health problems and multi-morbidity, and many different healthcare professionals are involved in the care for a patient. However, some healthcare professionals countered that this would complicate the issue to whom responsibility for referral to the LFO would belong.

I don’t think there’s really a good articulation whose job it is then, so if you’re doing it then you’re doing something extra. I’m quite willing to go for it, but I think people don’t necessarily perceive it as their job, they find it difficult. – Specialist (#33, neurology, age unknown)

### Stage 2: Care delivery at lifestyle front office

#### Financial burden additional visit

Patients mentioned that they would only go to the LFO if there were no additional costs involved. Many healthcare professionals confirmed this, and added that that it would hinder them in the process of referring patients if they knew patients had to pay for this additional care.

*If people need to pay for it themselves*,* then they might not do it [go to the LFO]. Or actually*,* I’m pretty sure that many people who are in my group of patients will not do it if that’s the case.* – Specialist (#17, internal medicine, 39 years old)

### Time, skills and knowledge of lifestyle broker

The LSB has more time to dedicate to patients (compared to other healthcare professionals) during appointment consultation, and this time can be used solely for discussing and guidance on lifestyle. The specific skills and knowledge that the LSB has (e.g. trained in Motivational Interviewing, knowledge on referral options and financial reimbursement policies) were seen as facilitators for implementation of an LFO.

I think a lot of doctors, but everyone actually, politicians too, vastly underestimate how difficult it is to bring about behavioural changes. If there are people who can look at that in a smart way, that would help tremendously. It is also true, of course, that it is not an open secret that smoking is not good for you. Yet it is very difficult to change people’s behaviour. Politics does little or nothing about it. I have never seen anyone quit after the doctor told them to stop smoking. So in that sense, such a person could be very helpful in encouraging or promoting good behaviour. – Specialist (#5, internal medicine, 41 years old)

Being interested in people, in their patients or clients. The lifestyle broker should enjoy their work, be skilful and not arrogant. And always talking with the patient. I do not see them as a doctor. You have a doctor and a coach. The coach is, it’s like the difference between a school teacher and the gym teacher, that’s how I see it. – Patient (#37, CVD, 52 years old)

Specifically their expertise and knowledge of the LSB on referral options was seen as facilitator for implementation, compared to the knowledge of other healthcare professionals in the hospital. In contrast, many healthcare professionals expressed concern that patients’ complex health requires specific support that only few community-based initiatives can provide. Some were doubtful that the LSB would be able to get in-depth knowledge on community-based initiatives, especially since the patient population in academic hospitals is spread over the country.

*I do wonder how you’re going to find out all the available options*,* because that must be an enormous amount. And then a part of me thinks*,* how are you going to find out if its evidence based?* – Nurse practitioner (#22, internal medicine, 59 years old)

#### Physical location of the LFO

Patients frequently mentioned that if the physical location of the LFO was placed far from the referring care department, it would lead to less compliance from both patients and healthcare professionals. Placing the LFO central to all hospital care would be beneficial for implementation.

[It would stop me from going to the lifestyle front office] If it’s really in a completely different location and I really have to go the other way. It would be nice if it’s just in one…. That it’s right next door – Patient (#34, CVD, 34 years old)

#### Efficiency in care planning

The level of efficiency in care planning was deemed as an important facilitator by both patients and healthcare professionals. It would facilitate patients in going to the LFO if waiting times were short, or if open consultation was a possibility. If appointments would be scheduled consecutively to other hospital appointments, this would also facilitate participation by patients. The possibility to schedule a telephone or video consultation was also seen as a facilitator.

Appointments at the LFO need to be combined with other appointments they [patients] have at the hospital. Sometimes people need to travel for more than an hour. You don’t want them to come here only for that appointment, they need to be able to combine it, or to have a video call instead. – Nurse practitioner (#23, internal medicine, 29 years old)

#### Fragmentation

Fragmentation of healthcare by adding a new component was mentioned by a few healthcare professionals as barrier. Contrarily, some healthcare professionals suggested that the LSB could function as case manager, connecting different stakeholders in the treatment process. This was deemed a facilitator.

I think the idea is very good, but, I have a slight “but”: every new idea has the pitfall that it fragmentises the [healthcare] lines, which are already pretty fragmented – Specialist (#20, internal medicine, 42 years old)

#### Prevention is a task of general practitioners

Some healthcare professionals and patients felt that general practitioners (GPs) are the responsible party when it comes to prevention-related healthcare, instead of hospitals. One healthcare professional thought it would be a good thing to join the trend of getting prevention out of the hospital and into the community by means of an LFO.

The advantage is that people listen to a doctor in a white coat, but the disadvantage is that healthy lifestyle is not necessarily something that belongs in a hospital. It would be better positioned by a GP, or I don’t know, somewhere in your neighbourhood or at a sport centre. – Patient (#59, osteoarthritis, 56 years old)

### Stage 3: Referral to community-based lifestyle initiative

#### Financial burden of community-based lifestyle initiative

The financial burden that could come with the community-based lifestyle initiatives was for many patients and almost all healthcare professionals mentioned as a barrier to go or refer to lifestyle initiatives.

*[.] and then they [doctor] said: ‘you should just get a personal trainer’. And that is their lifestyle advice. And I thought: personal trainer*,* that’s talking from privilege. [.] I don’t think they understand what that amount of money means to me. – Patient (#58*,* osteoarthritis*,* 52 years old)*.

The problem is that our perioperative, but especially postoperative care, is not covered by basic insurance when you look at physical therapy, for example. And that’s something that a lot of patients struggle with. And if you have to pay for it all by yourself, then it’s quite a cost, of course. So unlike other major surgeries, that’s not reimbursed with us and that’s tricky – Specialist (#3, internal medicine/nephrology, 46 years old)

#### Geographical availability

Additionally, almost all patients reported the geographical availability (or lack thereof) of interventions as a potentially inhibiting factor. Patients mentioned that if they would need to travel long distances, this would lead to non-compliance. The definition of ‘too far’ varied between patients from 20 min to several hours.

An hour [traveling to a physical therapist or dietician, for example] is the max though – Patient (#39, CVD, 34 years old)

#### Quality assurance of community-based lifestyle initiative

Knowledge at community-based lifestyle initiatives was seen as facilitating when the healthcare professionals in the community would be able to get in-depth knowledge on patients and their context. If they are able to identify complex health problems and appropriate interventions, this too was mentioned as facilitator. Some healthcare professionals, however, expressed their doubt about the quality assurance of the community-based lifestyle initiatives. Due to the variety in potentially fitting initiatives for patients, there is not one standard quality assurance system. A few specialists brought up that complex health problems require expertise and insufficient support may have dire consequences.

I think if you have someone who has more time for that to really guide people, and is more knowledgeable about that; has more tools to do that, that that could be a big thing; if the patient is motivated – Resident (#25, internal medicine, 30 years old)

See, there are patients with regular ‘garden-variety’ cardiology who can go to a regular gym. But when your cardiology problem is more the tertiary-care kind, you simply cannot go to the gym around the corner and get appropriate guidance and support – Patient (#42, CVD, 39 years old)

#### Collaboration

Collaboration between all healthcare professionals involved (working at the hospital, the community-based lifestyle initiative and general practice) was deemed important by healthcare professionals for implementation: lacking communication with primary care could lead to contradictory advice to patients. Continuous collaboration between all healthcare professionals and patients was regarded as necessary in order to support patients in securing information at the community-based lifestyle initiative and to participate, or to potentially return to the LFO to find a suitable alternative, if needed.

Ultimately, I would think of it as a large treatment team, where together we can encourage health-promoting behaviors – Specialist (#5, internal medicine, 41 years old)

### Implementation strategies

Based on the outcomes of the CFIR-ERIC matching tool we selected implementation strategies and operationalized them by combining strategies with practical suggestions from participants. Since only barriers can be matched to strategies with the tool, for facilitators we systematically reviewed the strategies identified through the matching process and assessed their relevance to the facilitators based on logical alignment and input from participants. Table 2 presents an overview of all implementation strategies and linkage with barriers and facilitators for implementation. To enhance clarity and readability, we chose to group similar or thematically related implementation strategies under overarching categories. This approach allows for a more coherent presentation within the context of this paper. See Appendix III for the complete overview of barriers, CFIR constructs, ERIC strategies and operationalized strategies.

*Build a viable infrastructure*. This strategy is proposed to ensure a viable (digital) infrastructure for the LFO. This infrastructure could involve digital resources (e.g. Electronic Patient Files (for documentation), hybrid consultation options, electronic referral to the LFO, and transmural electronic referral) and physical resources (e.g. physical location for the LFO, recruitment material).

*Create a learning collaborative*. Creating a learning collaborative network will improve collaboration among different stakeholders (especially transmural), facilitate information and experience exchange, and the possibility to learn and improve. Learning networks can be organised between LFOs, between LSBs, and with LFOs and the wider community.

*Prepare a referral tool with high quality referral options.* Developing a (digital) referral tool will provide knowledge on geographical availability of different referral options, which can be used to inform the patients of multiple available options nearby and identify the lack thereof. A referral tool should consider national quality control and quality assurance guidelines and fulfilment of formal caregivers, and be continuously updated with LSBs experiences with both formal and informal caregivers.

*Identify and prepare champions.* For the implementation of the LFO, selecting a dedicated healthcare professional (‘local champion’) at each department could support the innovation. This professional acts as a spokesperson for the LFO to their peers and facilitates in the organisation of meetings about the LFO.

*Inform and educate stakeholders about the LFO.* For healthcare professionals and board of directors of the hospital, this could initially be promoted through a department-wide email, information and educational presentations during staff meetings, informational videos, promotion materials (e.g. flyers, pocket cards with conversation starters) and consequently be covered by word of mouth among colleagues. For patients, this could be informing the client council and informing individual patients who have an appointment in the hospital through informative materials (e.g. waiting room screen, flyers, posters). For the community (i.e. GPs and community-based lifestyle initiatives) this could initially be through an email or presentation to the federation or networks of different formal caregivers and continuously through individual emails after a referral of a patient to the GP and community-based lifestyle initiative. And to reach a large group of stakeholders a newsletter can be send on a frequent basis. In all communication information regarding appurtenant procedures (e.g. how to refer a patient to the LFO, provided care in the LFO) and on less tangible matters (e.g. how to motivate patients) could be part of these information strategies.

*Conduct ongoing training*. For LSBs, ongoing training (i.e. training session, one-on-one coaching, intervision) could be developed on appurtenant procedures (i.e. how to refer patients to different community-based lifestyle initiatives, reporting in medical file, registration of care), but also on skills (e.g. motivational interviewing, lifestyle medicine knowledge) and knowledge about availability of referral options in the community with different financial reimbursement structures. The training can be supported by practical tools (e.g. manual, informational online platform).

*Build a coalition and promote network weaving*. Relationships with stakeholders are important to address factors within the hospital (i.e. feedback after referral, efficiency of care planning, skills and knowledge of healthcare professionals and of LSBs) and community referral related factors (i.e. collaboration, referral by GP for reimbursement, knowledge on referral options, geographical availability and quality assurance of community). A practical application in the hospital is that LSBs could join multidisciplinary staff meetings and come into contact with other healthcare professionals. Building relationships with community-based lifestyle initiatives and GPs could facilitate the creation of a smooth and efficient referral protocol. Relationships with healthcare insurance could be necessary for reimbursement of care delivery in the LFO and for autonomous possibilities for referral from the LFO (i.e. without involvement and additional burden of the GP).

*Collect testimonials of patient and healthcare professional experiences*. Success stories of patients who were referred to the LFO and from healthcare professionals could be captured in video and written word, and used as testimonials during (educational) stakeholder meetings, on the website and social media.

*Access new funding*. To address the barriers of financial burden, a financial structure needs to be designed on how the LFO is reimbursed as part of care as usual. On a practical level this means investment in partnerships with potential funding partners (e.g. healthcare insurers), and assessing funding options that already exist, in collaboration with stakeholders, such as the municipality, insurance companies, hospital procurement departments and governmental institutions. Furthermore, contacts with governmental institutions should be used to work on the introduction of a policy for the regular reimbursement of lifestyle interventions.

**Table 2:**
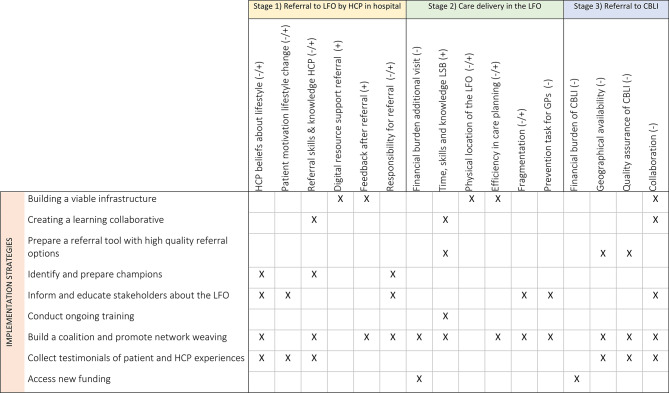
Barriers, facilitators and matching implementation strategies. - : barrier; + : facilitator

Abbreviations: HCP: Healthcare professional; LFO: lifestyle front office; GP: general practitioner; CBLI: Community-based lifestyle initiative

## Discussion

This study outlines key barriers, facilitators, and strategies for implementing the Lifestyle Front Office (LFO) within hospital care pathways. Identified barriers and facilitators related to referral include healthcare professionals’ beliefs, patient motivation, referral skills, digital support, feedback mechanisms, and role clarity. For attending the LFO and Lifestyle Support Base (LSB), factors include financial and time constraints, location, care coordination, and the GP’s role in prevention. Community-based initiative challenges include cost, accessibility, quality assurance, and collaboration. To address these, nine implementation strategies were proposed, such as building infrastructure, creating learning collaboratives, developing referral tools, training, stakeholder engagement, and securing funding. These results show how operationalized implementation strategies came to be and how they can assist in the integration of an LFO in a hospital. Even though more quotes from healthcare professionals are shown than quotes from patients, we value both perspectives equally as important and we attribute this imbalance to there being more themes relating to healthcare professionals and their respective tasks, work environment and skills in particular.

We chose to begin with an inductive analysis to gain a comprehensive understanding of all relevant themes related to the implementation of an LFO. While a deductive approach might have led to similar findings, it could have limited our ability to explore themes beyond the scope of the CFIR framework. At the time of the study, there were no known concepts similar to the LFO, so we wanted to remain open and thorough in our exploration. Conducting a thematic analysis enabled us to capture these differing perspectives, opinions, and implementation suggestions. In addition to our thematic analysis, we utilized the CFIR framework and its associated ERIC matching tool to identify suitable implementation strategies. Combining these approaches allowed for grounding our implementation plan in both data-driven insights and established theoretical guidance. The thematic analysis ensured that our understanding of barriers, facilitators, and stakeholder perspectives was not limited by predefined categories. This open-ended exploration provided context-specific themes that may not have emerged through a strictly deductive lens. By first conducting an inductive analysis and only then applying CFIR and ERIC, we were able to align our findings with a structured implementation science framework without losing the nuances of the data. Skipping directly to the matching tool would have risked overlooking important contextual factors and emerging themes that are critical for tailoring an effective implementation strategy. The combined approach strengthened the relevance, comprehensiveness, and applicability of our implementation plan. Although the CFIR-ERIC matching tool is designed to align barriers with appropriate implementation strategies, our findings also included a number of facilitators, which are often conceptually the inverse of barriers. Rather than disregarding these facilitators, we took a reflective approach by reviewing the strategies identified through the matching process and considering their potential to also reinforce or amplify existing strengths.

Studies on perspectives of healthcare professionals on implementing lifestyle and physical activity programs showed similar findings with regards to the attitude and beliefs of healthcare professionals, and the role of knowledge and skills in addressing lifestyle and suitable lifestyle interventions [32–34]. Among different settings, it has been recognized that both (lack of) motivation in patients and availability of resources related to (digital) communication and coordination are major barriers for adoption and implementation of healthcare innovation [33–36].

Additional expenses were mentioned in comparable Dutch studies: healthcare professionals are reluctant when it comes to additional healthcare of which it is not clear who will bear the costs (i.e. health insurance or patients) [14, 32]. Time constraints regarding addressing lifestyle and subsequent actions is a recurring barrier among many healthcare professionals in different settings [14, 32–36].

Financial accessibility of a lifestyle intervention is a concern for healthcare professionals, as they mostly do not want to increase costs for their patients. Additionally, the lack of knowledge on referral options and possibilities for reimbursement may enhance this [14, 32]. Concerns on quality of healthcare provided in the community and whether it is up to standard for their patients was also found in different studies related to implementing lifestyle-related healthcare [14, 32, 34]. Lastly, similar research has shown that insufficient collaboration between different levels of care, healthcare organizations, and professionals is by many deemed as a major barrier for implementation [32–34, 36].

Similar implementation strategies are seen in the literature regarding implementation of health innovations, such as use of champions [36], feedback and training (materials) [37]. Previous research has shown that important conditions for implementing healthcare innovations include the role of previous evidence of effectiveness and feasibility, the impact of contextual factors, and the impact of local champions and early adopters [38–40]. The involvement of stakeholders and positioning the patient at the centre of the care process are important principles for both design and implementation of the LFO [25, 41].

### Strength and limitations

This is to our knowledge the first study to investigate implementation challenges and strategies that accompany the introduction of an LFO in an outpatient hospital clinic. A strength of the current study is the sample size (*n* = 60), which is large for a qualitative study. The amount of interviewers (*n* = 7) may have affected data collection and analysis. Differences in experience (senior vs. intern) and interview styles may introduce some uncertainty in quality. On the other hand, we used a clear protocol for analysis and did these together which ultimately may have diminished the variation and any inconsistencies in quality. In the context of stakeholder involvement, this study provides a strong basis with input from both patients and a variety of healthcare professionals from different professions. However, all stakeholders were working or under treatment in an academic hospital and might as such not represent the care delivery in general hospitals. Furthermore, this study collected data within the hypothetical context of a future LFO, which limited the actual experience participants had. On the other hand, this offered an unique opportunity to adequately map implementation strategies to prospectively identified barriers and facilitators, which will be beneficial to initiating the LFO.

## Conclusions

This study gives insight in factors that facilitate or hinder implementation, and operationalisation of implementation strategies that can be used to set up an LFO or similar lifestyle medicine health services. However, future studies should investigate the proposed implementation strategies on effectiveness and impact on and fit with their context. More research is also needed regarding different patient populations, as different health conditions and treatments may affect perspectives and needs of lifestyle interventions, and of other healthcare settings (e.g. general hospitals, rehabilitation clinics). The results of this study are thought to be applicable to other hospitals, though implementation strategies are expected to need tailoring to the respective setting of the specific healthcare institute.

## Supplementary Information

Below is the link to the electronic supplementary material.

Supplementary Material 1

Supplementary Material 2

Supplementary Material 3

## Data Availability

The data supporting the findings of the study will be available upon reasonable request.

## Abbreviations

CBLI: Community-based lifestyle initiative
CFIR: Consolidated Framework for Implementation Research
EPF: Electronic Patient File
ERIC: Expert Recommendations for Implementing Change
GP: General practitioner
HCP: Healthcare professional
LFO: Lifestyle front office
LSB: Lifestyle broker
MI: Motivational Interviewing
NCD: Non-communicable diseases

## References

[1] Collaborators GRF. Global, regional, and National comparative risk assessment of 79 behavioural, environmental and occupational, and metabolic risks or clusters of risks, 1990–2015: a systematic analysis for the global burden of disease study 2015. Lancet (London England). 2016;388(10053):1659. 27733284 10.1016/S0140-6736(16)31679-8 PMC5388856

[2] WHO. Fact sheet: noncommunicable diseases [Online Fact Sheet]. Geneva: World Health Organisation; 2021. [updated 13-04-2021; cited 2021 02-08-2021].

[3] Volksgezondheidenzorg.info. Chronische aandoeningen en multimorbiditeit - cijfers en context: Huidige situatie [Webpage]. 2021 [updated 07-01-2021; cited 2021 08–08].

[4] Ezzati M, Riboli E. GLOBAL HEALTH behavioral and dietary risk factors for noncommunicable diseases. New Engl J Med. 2013;369(10):954–64. 24004122 10.1056/NEJMra1203528

[5] WHO. Global action plan for the prevention and control of NCDs 2013–2020. Geneva: World Health Organisation; 2013.

[6] Franz MJ, Boucher JL, Rutten-Ramos S, VanWormer JJ. Lifestyle weight-loss intervention outcomes in overweight and obese adults with type 2 diabetes: a systematic review and meta-analysis of randomized clinical trials. J Acad Nutr Dietetics. 2015;115(9):1447–63. 10.1016/j.jand.2015.02.031 25935570

[7] Johansen MY, MacDonald CS, Hansen KB, Karstoft K, Christensen R, Pedersen M, et al. Effect of an intensive lifestyle intervention on glycemic control in patients with type 2 diabetes: a randomized clinical trial. JAMA. 2017;318(7):637–46. 28810024 10.1001/jama.2017.10169 PMC5817591

[8] Saslow LR, Mason AE, Kim S, Goldman V, Ploutz-Snyder R, Bayandorian H, et al. An online intervention comparing a very low-carbohydrate ketogenic diet and lifestyle recommendations versus a plate method diet in overweight individuals with type 2 diabetes: a randomized controlled trial. J Med Internet Res. 2017;19(2):e5806. 10.2196/jmir.5806 PMC5329646 28193599

[9] O’Brien MJ, Perez A, Scanlan AB, Alos VA, Whitaker RC, Foster GD, et al. PREVENT-DM comparative effectiveness trial of lifestyle intervention and metformin. Am J Prev Med. 2017;52(6):788–97. 28237635 10.1016/j.amepre.2017.01.008 PMC5438762

[10] Freisling H, Viallon V, Lennon H, Bagnardi V, Ricci C, Butterworth AS et al. Lifestyle factors and risk of multimorbidity of cancer and cardiometabolic diseases: a multinational cohort study. BMC Med. 2020;18(1). 10.1186/s12916-019-1474-7 PMC6953215 31918762

[11] Jarbol DE, Larsen PV, Gyrd-Hansen D, Sondergaard J, Brandt C, Leppin A, et al. Determinants of preferences for lifestyle changes versus medication and beliefs in ability to maintain lifestyle changes. A population-based survey. Prev Med Rep. 2017;6:66–73. 28271023 10.1016/j.pmedr.2017.02.010 PMC5331161

[12] Van Zaanen Y, Hoorntje A, Koenraadt KLM, Van Bodegom-Vos L, Kerkhoffs GMMJ, Waterval-Witjes S, et al. Non-surgical treatment before hip and knee arthroplasty remains underutilized with low satisfaction regarding performance of work, sports, and leisure activities. Acta Orthop. 2020;91(6):717–23. 32878525 10.1080/17453674.2020.1813440 PMC8023969

[13] Alageel S, Gulliford MC, McDermott L, Wright AJ. Implementing multiple health behaviour change interventions for cardiovascular risk reduction in primary care: a qualitative study. BMC Fam Pract. 2018;19. 10.1186/s12875-018-0860-0 PMC6208114 30376826

[14] Nauta J, van Nassau F, Bouma AJ, Krops LA, van der Ploeg HP, Verhagen E, et al. Facilitators and barriers for the implementation of exercise are medicine in routine clinical care in Dutch university medical centres: a mixed methodology study on clinicians’ perceptions. BMJ Open. 2022;12(3):e052920. 35292491 10.1136/bmjopen-2021-052920 PMC8928323

[15] Jallinoja P, Absetz P, Kuronen R, Nissinen A, Talja M, Uutela A, et al. The dilemma of patient responsibility for lifestyle change: perceptions among primary care physicians and nurses. Scand J Prim Health. 2007;25(4):244–9. 10.1080/02813430701691778 PMC3379767 17934984

[16] Hamilton K, Henderson J, Burton E, Hagger MS. Discussing lifestyle behaviors: perspectives and experiences of general practitioners. Health Psychol Behav. 2019;7(1):290–307. 10.1080/21642850.2019.1648216 PMC8114406 34040852

[17] Clark RE, McArthur C, Papaioannou A, Cheung AM, Laprade J, Lee L, et al. I do not have time. Is there a handout I can use? Combining physicians’ needs and behavior change theory to put physical activity evidence into practice. Osteoporos Int. 2017;28(6):1953–63. 28413842 10.1007/s00198-017-3975-6

[18] Thijssen SV, Jacobs MJG, Swart RR, Heising L, Ou CXJ, Roumen C. The barriers and facilitators of radical innovation implementation in secondary healthcare: a systematic review. J Health Organ Manag. 2021. 10.1108/JHOM-12-2020-0493 PMC10430798 34910413

[19] Peters DH, Tran NT, Adam T. Implementation research in health: a practical guide. World Health Organization; 2013.

[20] Peters DH, Adam T, Alonge O, Agyepong IA, Tran N. Implementation research: what it is and how to do it. BMJ: Br Med J. 2013;347:f6753. 24259324 10.1136/bmj.f6753

[21] Miller WRR. S. Motivational interviewing: Preparing people to change addictive behavior. New York: Guildford; 1991.

[22] VanBuskirk KA, Wetherell JL. Motivational interviewing with primary care populations: a systematic review and meta-analysis. J Behav Med. 2014;37(4):768–80. 23934180 10.1007/s10865-013-9527-4 PMC4118674

[23] Environment NIfPHa. Organisation of Health Care [Available from: https://www.rivm.nl/zorg/organisatie-van-zorg

[24] Argumentenfabriek D. This is How Healthcare Works [Available from: https://www.zowerktdezorg.nl/home-english/

[25] te Loo LM, Holla JFM, Vrijsen J, Driessen A, van Dijk ML, Linders L et al. Implementation barriers and facilitators for referral from the hospital to community-based lifestyle interventions from the perspective of lifestyle professionals: a qualitative study. PLoS ONE. 2024;19(6). 10.1371/journal.pone.0304053 PMC11210764 38935601

[26] Tong A, Sainsbury P, Craig J. Consolidated criteria for reporting qualitative research (COREQ): a 32-item checklist for interviews and focus groups. Int J Qual Health Care. 2007;19(6):349–57. 17872937 10.1093/intqhc/mzm042

[27] Flottorp SA, Oxman AD, Krause J, Musila NR, Wensing M, Godycki-Cwirko M et al. A checklist for identifying determinants of practice: a systematic review and synthesis of frameworks and taxonomies of factors that prevent or enable improvements in healthcare professional practice. Implement Sci. 2013;8. 10.1186/1748-5908-8-35 PMC3617095 23522377

[28] B.V. AG. 2024 Available from: https://www.amberscript.com/en/

[29] Castleberry A, Nolen A. Thematic analysis of qualitative research data: is it as easy as it sounds? Curr Pharm Teach Learn. 2018;10(6):807–15. 30025784 10.1016/j.cptl.2018.03.019

[30] Damschroder LJ, Aron DC, Keith RE, Kirsh SR, Alexander JA, Lowery JC. Fostering implementation of health services research findings into practice: a consolidated framework for advancing implementation science. Implement Sci. 2009;4. 10.1186/1748-5908-4-50 PMC2736161 19664226

[31] Powell BJ, Waltz TJ, Chinman MJ, Damschroder LJ, Smith JL, Matthieu MM, et al. A refined compilation of implementation strategies: results from the expert recommendations for implementing change (ERIC) project. Implement Sci. 2015;10:21. 25889199 10.1186/s13012-015-0209-1 PMC4328074

[32] IJsbrandy C, van Harten WH, Gerritsen WR, Hermens RPMG, Ottevanger PB. Healthcare professionals’ perspectives of barriers and facilitators in implementing physical activity programmes delivered to cancer survivors in a shared-care model: a qualitative study. Support Care Cancer. 2020;28(7):3429–40. 31792881 10.1007/s00520-019-05108-1 PMC7256088

[33] Bouma SE, van Beek JFE, Alma MA, Diercks RL, van der Woude LHV, van den Akker-Scheek I, et al. What affects the implementation of lifestyle interventions in patients with osteoarthritis? A multidisciplinary focus group study among healthcare professionals. Disabil Rehabil. 2022;44(26):8283–93. 34889696 10.1080/09638288.2021.2011438

[34] Kulmala J, Rosenberg A, Ngandu T, Hemio K, Tenkula T, Hyytia A, et al. Facilitators and barriers to implementing lifestyle intervention programme to prevent cognitive decline. Eur J Public Health. 2021;31(4):816–22. 34448856 10.1093/eurpub/ckab087 PMC8505000

[35] Smock C, Alemagno S. Understanding health care provider barriers to hospital affiliated medical fitness center facility referral: a questionnaire survey and semi structured interviews. Bmc Health Serv Res. 2017;17(1):520. 28774290 10.1186/s12913-017-2474-y PMC5543749

[36] Krska J, du Plessis R, Chellaswamy H. Views of practice managers and general practitioners on implementing NHS health checks. Prim Health Care Res. 2016;17(2):198–205. 10.1017/S1463423615000262 25991495

[37] Dryden-Palmer K, Berta WB, Parshuram CS. Implementing a complex hospital innovation: conceptual underpinnings, program design and implementation of a complex innovation in an international multi-site hospital trial. Bmc Health Serv Res. 2022;22(1). 10.1186/s12913-022-08768-8 PMC9652896 36371214

[38] Barnett J, Vasileiou K, Djemil F, Brooks L, Young T. Understanding innovators’ experiences of barriers and facilitators in implementation and diffusion of healthcare service innovations: a qualitative study. Bmc Health Serv Res. 2011;11. 10.1186/1472-6963-11-342 PMC3265424 22176739

[39] Robert G, Greenhalgh T, MacFarlane F, Peacock R. Adopting and assimilating new non-pharmaceutical technologies into health care: a systematic review. J Health Serv Res Po. 2010;15(4):243–50. 10.1258/jhsrp.2010.009137 20592046

[40] Fitzgerald L, Ferlie E, Hawkins C. Innovation in healthcare: how does credible evidence influence professionals? Health Soc Care Comm. 2003;11(3):219–28. 10.1046/j.1365-2524.2003.00426.x 12823426

[41] Parmar J, Sacrey LA, Anderson S, Charles L, Dobbs B, McGhan G, et al. Facilitators, barriers and considerations for the implementation of healthcare innovation: A qualitative rapid systematic review. Health Soc Care Comm. 2022;30(3):856–68. 10.1111/hsc.13578 34558143

