# Supplementary material for: Matching implementation strategies to barriers and facilitators for a lifestyle front office in the hospital: a qualitative study
Source: BMC Health Serv Res. 2025 Sep 30;25:1241. doi: 10.1186/s12913-025-13452-8 (PMC12482734; doi:10.1186/s12913-025-13452-8)
Supplement: Supplementary file 2 — Supplementary Material 2 [file 12913_2025_13452_MOESM2_ESM.docx]

**Appendix II: Topic guide for patients**

| **Topic guide for interviewing patients**  Date: Initials interviewer:  Audiofile number:  *Introduction*  *Thank you very much for your participation in this study. My name is [...]. Today we will talk about a number of issues related to setting up a lifestyle front office. The LOFIT study is about setting up, implementing and evaluating a lifestyle front office within the hospital [name hospital]. By lifestyle front office we mean a place where patients are motivated to make lifestyle changes and are guided to suitable support to improve their lifestyle. By lifestyle we mean the behaviors you have developed in terms of diet, smoking, exercise, alcohol consumption, stress, sleep. The lifestyle broker, working in this supporting role, guides patient to local lifestyle initiatives that can help with lifestyle guidance. The goal of LOFIT is to optimize hospital care by making referral to initiatives in the region a regular part of regular care. This interview contributes to the scientific foundation of the lifestyle front office, and its design and implementation.*  *Before we begin, I would like to ask your permission to make an audio recording of this interview. The interview will last approximately 45 minutes. The interview will remain confidential; all data will be stored securely and encrypted. Upon completion of this interview, we will transcribe this audio recording and then delete it. <sign form informed consent>.*  *Do you have any further questions before we start?* | |
| --- | --- |
| *Demographic characteristics*  To begin, I would like to ask some general questions about yourself and how you ended up at [name hospital].   \| *Question* \| *Answer* \| \| --- \| --- \| \| What is your age? \| … year \| \| What is your gender? \| Man / Woman / Else \| \| What is your highest completed education? \|  \| \| What is your place of residence? \|  \| \| At which hospital are you based? \|  \| \| Which department/specialty are you familiar with? \|  \| \| How long have you been under treatment/monitoring at this hospital? \|  \| | |
| *Domain* | *Questions and prompts* |
| Personal situation health problem | - Can you tell something about your health problem/condition and how you ended up at Amsterdam UMC - VUmc/UMCG? - How many years have you been living with your health problem/   condition?  o Prompt: What was the reason for you to seek medical help? |
| Experience current situation regarding lifestyle and behavior change in hospital care | We know more and more about the importance of a healthy lifestyle and the effects on cardiovascular disease / osteoarthritis. By a healthy lifestyle we think of, sufficient exercise, healthy diet, moderate alcohol consumption, low stress, sufficient sleep, stop smoking.  - Do you think you could improve your lifestyle in any of these areas? (prompt: exercise, diet, alcohol, stress, sleep, smoking)  - Did your attending physician discuss your lifestyle during your hospital appointment, if so how? And what did you think of this; if not, would you have liked to? (prompt: why?; how?)  - Based on this conversation with your doctor, have you changed anything in your lifestyle?   o Or if previous question no: Have you changed anything about your lifestyle recently?  o If yes: What helped you do this? What hindered you? (Prompt: for each lifestyle behavior, ask about impeding and promoting factors)  o If no: What hinders you in making a behavior change (prompt: each lifestyle type) and what helps you or could help you in making a behavior change?  - Did your treating physician also discuss medication use with you? And in what way was this done: did you have a choice in this or was it an advice?  o If advice: what if the doctor had left the choice to you, what would you have chosen in order to get your disease under control: only lifestyle adjustments, only taking medication or a combination of both? (prompt: what plays a role in this consideration)  o If choice: what did you think at the time? / How did you make this choice then? (prompt: what played into this consideration) |
| Experience current situation regarding lifestyle and behavior change in hospital care /  Expectations and preferences of a lifestyle front office | - Overall, what is your assessment of the role lifestyle counseling has in your treatment/monitoring?  o Do you think it should receive more/less attention? Why?  We know from research that physicians have too little time to discuss patients' lifestyle in sufficient detail in a consultation, and for that reason we want to set up a lifestyle desk where specialists have the time and knowledge to discuss lifestyle and behavior change, but also where there is knowledge about referral options in your area.  - What do you think of this lifestyle front office idea? And would you go here yourself if offered this opportunity? (prompt: what would motivate you (e.g., the role of the doctor) and what would hold you back?)  - What do you think a lifestyle front office should offer you?  - If you are making a decision about your lifestyle or changing your lifestyle, how do you approach it? What information would you like to receive? (prompt: for different lifestyle changes - smoking, exercise, nutrition, sleep, stress, alcohol)  We would like such a lifestyle front office to be staffed by a lifestyle broker: this is a kind of lifestyle coach who can guide you but also further help you find suitable help or activities in your area.  - What would you consider important characteristics of such a lifestyle broker?  - How long do you think a conversation with such a lifestyle broker should take? And how often a year would you like to have contact with the lifestyle broker? (prompt: in what form - face to face / by phone / online)  - What would be topics you would like to discuss with the lifestyle broker? |
| Preferences and needs when referring to local lifestyle care | - - What do you think is important about the parties you are referred to? - o Prompt: Location, how would you like to reach it/how long may the travel distance be? - o Prompt: Would you prefer counseling in a a group or individually? |
| *Summarize the most important points mentioned.*  - Do you have any further additions or comments that we have not discussed, but are relevant *[important]* to the interview?  - Thank you very much for participating in this interview. We will take the results into account when setting up the lifestyle front office. | |

**Appendix III: Topic guide for healthcare professionals**

| **Topic guide for interviewing stakeholders – healthcare professionals**  Date: Initials interviewer:  Audio file nr:  *Introduction*  *Thank you very much for your participation in this study. My name is [...]. Today we are going to talk about a number of issues related to setting up a lifestyle front office. The LOFIT study is about setting up, implementing and evaluating a lifestyle front office within the hospitals Amsterdam UMC, location VUmc and AMC, and UMC Groningen. By lifestyle front office we mean a place where patients are helped to find suitable support to improve their lifestyle. By lifestyle we mean the behaviors you have developed in terms of diet, smoking, exercise, alcohol consumption, stress, sleep. The lifestyle broker, working in this supporting role, looks with the patient at local initiatives that can help them with this. The goal of LOFIT is to optimize hospital care by making referral to initiatives in the region a regular part of regular care. This interview contributes to the scientific foundation of the lifestyle front office and its design and implementation.*  *Before we begin, I would like to ask your permission to make an audio recording of this interview. The interview will last approximately 45 minutes. The interview will remain confidential; all data will be stored securely and encrypted. Upon completion of this interview, we will type out this audio recording and then delete it. <sign form informed consent>.*  *Do you have any further questions before we begin?* | |
| --- | --- |
| *Demographic characteristics*  To begin, let me ask some general questions about your position and your relationship to the hospital where you work.   \| *Question* \| *Answers* \| \| --- \| --- \| \| What is your age? \| … years \| \| What is your gender? \| Man / Woman/ Other \| \| At which (healthcare) institution are you employed? \|  \| \| What is your position? \|  \| \| How many years have you worked in this position? \| … year(s) \| \| What patient group do you primarily treat? \|  \| | |
| *Domain* | *Questions and prompts* |
| Experience current situation regarding lifestyle in hospital care | - In general, what is your assessment of the role of lifestyle advice in a patient's treatment/monitoring?  - Do you ever enter into the conversation with your patients about a healthier lifestyle? How do you approach that conversation at such times?  o prompt: what do you tell the patient? (including what advice is given, referral to guidelines and standards; referral to other healthcare providers or local initiatives (both inside and outside the hospital))  o prompt: in which patient group do you or would you like to have this conversation about a healthier lifestyle? And why exactly this group?  - What are your experiences so far with the patients with whom you have addressed "lifestyle" in the consultation?  o Prompt: experiences on the quality of professional (how easy is this for them to bring up: including what helps them and hinders them from having this conversation)  o Prompt: experiences on the effects on patient outcomes (including patient follow-through on appointments made; problems experienced by patient, effects on treatment) |
| Preferences and needs regarding lifestyle front office in hospital | Previous research has shown that as a physician you have little time to discuss patients' lifestyle in sufficient detail during a consultation. For this reason, we want to set up a lifestyle front office in the hospital where specialists have the time and knowledge to discuss lifestyle and behavioral changes, but also where there is knowledge about possible referral options in your region in order to guide the patient as best as possible in making a behavioral change in diet, smoking, exercise, stress, sleep, alcohol consumption.  - What is your initial reaction to this idea?  o Prompt: would you refer patients to the lifestyle front office? (including what would hinder you in this and what could help you in this).  - How would the lifestyle front office be embedded within existing treatment? Prompt: at what point in treatment do you refer patients to the lifestyle front office?    - In your opinion, what are the key parties/professionals in your department that should be considered in the design of a lifestyle front office? And why? Prompt: (colleagues; dieticians, nurses, ...)  We think it is important to provide feedback on the patient's progress to the attending physician.  - What are your thoughts on this?  o Prompt: what should this feedback include? (including how (digital - file - face to face); content (what should be included here); how often)  - Reference to previously mentioned patient group: Does this fit the patient group you mentioned earlier or are there other patient groups where you think guidance from a lifestyle broker could work positively? |
| Please summarize the main points raised.  - Do you have any further additions or comments that we have not discussed but are relevant to the interview?  - Thank you very much for your participation in this interview. We will take the results into account in the design and implementation of the lifestyle front office.  - Are there any other colleagues you think might be useful if we spoke with them?  - Would you like to be kept informed about this project - e.g. in a newsletter. | |
