# Supplementary material for: Matching implementation strategies to barriers and facilitators for a lifestyle front office in the hospital: a qualitative study
Source: BMC Health Serv Res. 2025 Sep 30;25:1241. doi: 10.1186/s12913-025-13452-8 (PMC12482734; doi:10.1186/s12913-025-13452-8)
Supplement: Supplementary file 3 — Supplementary Material 3 [file 12913_2025_13452_MOESM3_ESM.docx]

Appendix III: Overview of barriers, CFIR constructs, ERIC strategies and operationalization

|  | Barrier | CFIR constructs | ERIC strategies | Operationalized strategies |
| --- | --- | --- | --- | --- |
| Stage 1: Referral to LFO by HCP in hospital | HCP beliefs about lifestyle | Evidence strength & quality; Knowledge & beliefs about intervention; Tension for change; Relative advantage | Access new funding | Identify possibilities for potential partners in funding (municipality, board of directors, health insurance companies) |
|  |  |  | Conduct educational meetings | Instruct clinicians on referral procedures and patient journey, regularly join department meetings for instruction and updates |
|  |  |  | Inform local opinion leaders | Inform board of directors, department heads and managers about LFO and procedures |
|  |  |  | Assess for readiness and identify barriers and facilitators | Interview clinicians and other hospital workers (current study) |
|  | Patient motivation for lifestyle change | Patient/customers;  Tension for change;  Knowledge & beliefs about the intervention | Identify and prepare champions | Capture positive experiences and success stories of patients (testimonials) in written text, audio and video. |
|  |  |  | Conduct local consensus discussions | Instruct clinicians on who is eligible for referral and how to deal with unmotivated patients |
|  |  |  | Involve patients and family members | Inform patients about LFO, provide information material (flyers, posters, videos), website with information |
|  |  |  | Prepare patients to be active participants |  |
|  | Referral skills & knowledge HCP | Knowledge & beliefs about the intervention;  Access to knowledge & information | Build a coalition | Identify projects that relate to organization of healthcare and lifestyle, including academic projects; CBLIs; networks; umbrella organizations. |
|  |  |  | Develop academic partnerships |  |
|  |  |  | Promote network weaving |  |
|  |  |  | Visit other sites | Identify similar services, get in contact and plan visits |
|  | Responsibility for referral | Key stakeholders;  Individual stage of change; Structural characteristics;  Tension for change | Identify and prepare champions | Find ‘believers’ in departments to help convince non-believers of value of lifestyle advice and LFO |
|  |  |  | Conduct local consensus discussions | Organize meetings to emphasize importance, organization and responsibilities in LFO care pathway |
|  |  |  | Assess for readiness and identify barriers and facilitators | Interview clinicians and department heads (current study) |
|  |  |  | Inform local opinion leaders | Inform department heads and managers about LFO |
| Stage 2: Care delivery in the LFO | Financial burden additional visit | Costs; Available resources | Access new funding | Identify possibilities for reimbursement or funding (municipality, board of directors, health insurance companies) |
|  |  |  | Develop resource sharing agreements | Identify potential partners within domain and form partnerships (e.g. health insurance companies) |
|  |  |  | Fund and contract for clinical innovation | Explore/identify possibilities for sustainable embedding of LFO within healthcare system |
|  |  |  | Alter incentive/allowance structures | - |
|  | Physical location of the LFO;  Efficiency in care planning; Fragmentation * | Complexity;  Capability | Promote adaptability | Care delivery from LFO must be made available in hybrid mode (physical location and video consultation) |
|  |  |  | Tailor strategies | Appointments in LFO need to fit schedule (i.e. before or after other appointments in hospital); patient needs to be informed about LFO and appointment on beforehand; LFO needs to be central in hospital |
|  |  |  | Identify and prepare champions | Present LSB as case manager and bridge between different components of care pathway and stakeholders |
|  |  |  | Assess for readiness and identify barriers and facilitators | Interview clinicians and other hospital workers (current study) |
|  | Prevention task for GPs | Culture;  External change agents | Build a coalition | Build infrastructure to facilitate referral both inside and outside of the hospital (e.g. network platform), foster partnerships with GPs and provide them with information material |
|  |  |  | Involve executive boards | Engage with board of directors and patient federation and provide them with information on LFO and benefits for hospital patients |
|  |  |  | Use advisory boards and workgroups | Use consortium meetings (with different kinds of stakeholders) for input and advice on implementation |
|  |  |  | Identify and prepare champions | Identify enthusiastic GPs and engage with them, provide them with information in order to engage colleagues and patients. |
| Stage 3) Referral to CBLI | Financial burden of CBLI | Costs; Available resources | Access new funding | Explore possibilities for additional reimbursement/exemption conditions in municipalities |
|  |  |  | Fund and contract for clinical innovation |  |
|  |  |  | Alter incentive/allowance structures |  |
|  |  |  | Develop resource sharing agreements | Identify free and low-cost referral options for patients, form partnerships with similar initiatives to help with this exploration |
|  | Geographical availability | Available resources; patients/customers | Access new funding | Explore possibilities within municipality for residents to participate in lifestyle interventions, access to social services etc. |
|  |  |  | Fund and contract for clinical innovation |  |
|  |  |  | Change physical structure and equipment | Map CBLIs in regions, assess potential deficient areas and make partner organizations aware of lack of possibilities to help activate local parties. Make use of existing platforms. |
|  |  |  | Develop resource sharing agreements |  |
|  | Quality assurance of CBLI | Individual stage of change; external policy & incentives | Create a learning collaborative | Capture experiences and stories of patients (testimonials) in written text, audio and video; create shared learning environment for LSBs and CBLIs to communicate and share experiences. |
|  |  |  | Alter incentive/allowance structures | Create referral structure in which patients are referred to CBLIs that have proven to be of good quality and trustworthy; vice versa for negative experiences. Create system in which criteria can be noted and experiences be tracked; get consensus on quality requirements and design monitoring strategy. |
|  |  |  | Identify and prepare champions | Identify person within team who can monitor quality of CBLIs |
|  |  |  | Make training dynamic | Design training and referral tool for LSBs to provide skills in referring to CBLIs of high quality. |
|  | Collaboration | Complexity; compatibility; structural characteristics | Promote adaptability | - |
|  |  |  | Conduct local consensus discussions | Involve all stakeholders in concept of LFO, inform about start and provide information on goals, protocol and benefits. |
|  |  |  | Conduct cyclical small tests of change | - |
|  |  |  | Assess for readiness and identify barriers & facilitators | Start partnerships with stakeholders, stay in communication and evaluate periodically |

Abbreviations: HCP = Healthcare Professional; LFO = Lifestyle Front Office; CBLI = Community-based Lifestyle Initiative; LSB = Lifestyle Broker; GP = General Practitioner
* For barriers ‘physical location of the LFO’, ‘efficiency in care planning’ and ‘fragmentation’, CFIR themes and ERIC strategies were similar.
